# USP14 is a deubiquitinase for Ku70 and critical determinant of non-homologous end joining repair in autophagy and PTEN-deficient cells

**DOI:** 10.1093/nar/gkz1103

**Published:** 2019-11-19

**Authors:** Arishya Sharma, Turkeya Alswillah, Isha Kapoor, Pal Debjani, Belinda Willard, Matthew K Summers, Zihua Gong, Alexandru Almasan

**Affiliations:** 1 Department of Cancer Biology, Cleveland Clinic, 9500 Euclid Avenue, Cleveland, OH 44195, USA; 2 Department of Chemistry, Cleveland State University, 2121 Euclid Avenue, Cleveland, OH 44115, USA; 3 Department of Radiation Oncology and the Comprehensive Cancer Center, The Ohio State University, Columbus, OH 43017, USA; 4 Proteomics and Metabolomics Core, Lerner Research Institute, Cleveland Clinic, 9500 Euclid Avenue, Cleveland, OH 44195, USA; 5 Department of Radiation Oncology, Taussig Cancer Institute, Cleveland Clinic, 9500 Euclid Avenue, Cleveland, OH 44195, USA; 6 Case Comprehensive Cancer Center, Case Western Reserve University School of Medicine, Cleveland, OH 44106, USA

## Abstract

Ionizing radiation (IR)-induced DNA double-strand breaks (DSBs) are predominantly repaired by non-homologous end joining (NHEJ). IR-induced DNA damage activates autophagy, an intracellular degradation process that delivers cytoplasmic components to the lysosome. We identified the deubiquitinase USP14 as a novel autophagy substrate and a regulator of IR-induced DNA damage response (DDR) signaling. Inhibition of autophagy increased levels and DSB recruitment of USP14. USP14 antagonized RNF168-dependent ubiquitin signaling and downstream 53BP1 chromatin recruitment. Here we show that autophagy-deficient cells are defective in NHEJ, as indicated by decreased IR-induced foci (IRIF) formation by pS2056-, pT2609-DNA-PKcs, pS1778-53BP1, RIF1 and a reporter assay activation. Moreover, chromatin recruitment of key NHEJ proteins, including, Ku70, Ku80, DNA-PKcs and XLF was diminished in autophagy-deficient cells. USP14 inhibition rescued the activity of NHEJ-DDR proteins in autophagy-deficient cells. Mass spectrometric analysis identified USP14 interaction with core NHEJ proteins, including Ku70, which was validated by co-immunoprecipitation. An in *vitro* assay revealed that USP14 targeted Ku70 for deubiquitination. AKT, which mediates Ser432-USP14 phosphorylation, was required for IRIF formation by USP14. Similar to USP14 block, AKT inhibition rescued the activity of NHEJ-DDR proteins in autophagy- and PTEN-deficient cells. These findings reveal a novel negative PTEN/Akt-dependent regulation of NHEJ by USP14.

## INTRODUCTION

Radiotherapy (RT) is a highly effective treatment modality for local control of many, if not most, cancer histologies. While RT eradicates tumors by inducing lethal DNA double-strand breaks (DSBs) in cells, tumor cell DSB repair pathways contribute to resistance against the treatment. Therefore, uncovering novel mechanisms that can limit or antagonize cancer cell DSB repair holds promise to enhance effectiveness of RT to control tumor cell growth and survival (1).

Two major pathways are employed by eukaryotic cells for the repair of DSBs, non-homologous end joining (NHEJ) and homologous recombination (HR). NHEJ is active throughout the cell cycle and is, therefore, the major pathway choice responsible for DSB repair (2). In contrast, HR depends on the presence of a sister chromatid as a template and is, therefore, restricted to late S- and G2-phases of the cell cycle (3). Thus, an appropriate pathway choice is tightly regulated through the cell cycle of both normal and cancer cells to maintain cellular genomic stability. Ubiquitination of histone H2A by E3 ligases RNF8 and RNF168 plays an important part in DNA repair pathway choice by recruiting 53BP1 to DSBs. 53BP1, together with its partner proteins RIF1 (Rap1-interacting factor 1) and PAX transcription activation domain interacting protein (PTIP), inhibits Breast Cancer gene 1 (BRCA1)–CTBP interacting protein (CtIP) complex-dependent DSB end resection (4). This promotes rapid NHEJ of the DSB ends and inhibits the HR pathway. Classical NHEJ involves sensing and binding of the Ku70/Ku80 heterodimer to DNA DSBs, with subsequent recruitment of DNA-dependent protein kinase, catalytic subunit (DNA-PKcs) and end-processing factors leading to repair by the DNA ligase IV/X-ray repair cross-complementing protein 4 (XRCC4)/XRCC4-like factor (XLF) complex (2).

In response to DNA damaging agents, including ionizing radiation (IR), cancer cells activate autophagy as a means to remove damaged organelles and protein aggregates to promote overall survival (5). However, autophagy may serve as a pro-death or -survival pathway in response to IR-treatment depending on cellular context (6,7). Clearly, a better understanding of the cross-talk between autophagy and DSB repair pathways will enable us to identify molecular determinants of cellular response to manipulating autophagy in the context of radiosensitivity. Interestingly, in recent years autophagy has emerged as an important determinant of DSB repair process. Autophagy has been shown to regulate the levels of critical DDR-associated proteins, including checkpoint kinase 1 (CHEK1/CHK1) (8), Sae2, the yeast homolog of CtIP (9) and CBX/HP1 (10). Moreover, autophagy has been shown to promote HR through inhibition of proteasomal degradation of filamin A and RAD51 (11), and activation of RNF168 (12). While these various studies have addressed the regulation of HR by autophagy, there are no studies on how autophagy impacts NHEJ, the major DSB repair pathway for IR-induced DSBs.

We have recently identified USP14 as a critical negative regulator of RNF168 protein expression and RNF168-associated ubiquitin (Ub) signaling in response to IR (13). In addition, we revealed that USP14 is degraded through autophagy. Thus, in autophagy-deficient cells, increased levels of USP14 led to inhibition of RNF168 and 53BP1 IR-induced foci (IRIF) formation (13). While our previous findings imply a connection between autophagy and NHEJ through the 53BP1/RNF168 axis, a clear effect on NHEJ pathway *per se* has not been investigated.

USP14 is a major regulator of the proteasome, and one of three proteasome-associated DUBs (14,15). USP14 promotes Ub recycling (16,17). In addition to this catalytic role, USP14 is a major allosteric regulator of proteasome function that has the unusual capacity to act at multiple steps in substrate degradation (17). USP14 depletion is known to modulate substrate protein levels as well as decrease available free Ub pools (18). USP14 is known to promote oncogenesis in several tumor types, and pharmacological inhibition of USP14 has been shown to effectively control tumor growth (19,20). However, to date there is only a preliminary account of its endogenous substrates and its potential to regulate the DNA damage response (DDR) is largely unknown.

Phosphatase and tensin homolog (PTEN) is the most frequently mutated or deleted gene in prostate cancer (PCa) (21). Loss of PTEN gene leads to subsequent activation of AKT, a serine/theronine kinase, is a critical event in PCa (22). It has been reported that AKT mediates phosphorylation of USP14 on Ser432 (23). However, the physiological relevance of AKT-dependent activation of USP14 in the nucleus has not been examined.

Here we show that USP14 directly regulates NHEJ repair. Our mass spectrometric analysis identified USP14 interaction with critical core NHEJ proteins. In addition, autophagy-deficient cells manifested defects in NHEJ signaling, as shown by decreased IRIF formation by pS2056- and pT2609-DNA-PKcs, pS1778 53BP1 (a substrate for DNA-PKcs) and RIF1. Moreover, chromatin recruitment of key NHEJ regulatory proteins, including 53BP1, Ku70 and XRCC4 was diminished in autophagy-deficient cells which could be rescued by USP14 inhibition or knockdown. Thus, USP14 emerges as a critical determinant of NHEJ repair response by both directly targeting NHEJ core assembly and antagonizing RNF168-dependent ubiquitination signaling. Importantly, we show that the DSB-repair function of USP14 is dependent on the constitutive AKT activation found in advanced tumors that are PTEN deficient.

## MATERIALS AND METHODS

### Cell culture and treatments

shATG7 inducible LNCaP cells were a kind gift from Dr Daniel E. Frigo (U Texas MD Anderson Cancer Center) (24). VCaP cells were a kind gift from Dr. Hannelore Heemers (Cleveland Clinic Lerner Research Institute). Cells were maintained in RPMI medium containing L-glutamine, supplemented with 10% fetal bovine serum, and 100× antibiotic-antimycotic. HEK 293T cells were maintained in DMEM. Cells were grown in a humidified incubator at 37°C and 5% CO_2_.

Cells were irradiated with 10 Gy, unless otherwise mentioned, at 25°C, using a Mark I Irradiator (J.C. Shepherd & Associates, Irvine, CA, USA) with a ^137^Cs source emitting at a fixed dose-rate of 2.0 Gy/min, as described previously (13). Cells were treated with 50 μM IU1 (Selleck, Houston, TX, USA), 10 μM CQ (Sigma-Aldrich), 500 nM Nu7441 (Selleck) and 1 μM MK2206 (Selleck), wherever mentioned.

### Plasmids

The GFP-PTEN plasmid was a kind gift from Dr Charis Eng (Lerner Research Institute, Cleveland Clinic Foundation) Flag-HA-KU70 (46957) and Flag-HA-USP14 (22569) were from Addgene (Watertown, MA, USA). USP14 (TRCN0000007426) short-hairpin RNA (shRNA)-expressing lentiviral plasmids were made in pLKO.1-puro and purchased from Sigma-Aldrich (St. Louis, MO, USA). The lentiviral packaging plasmids pCMV-VSV-G and pCMV-GAP-POL were from Sigma-Aldrich.

### Lentiviral transduction of shRNA

HEK 293T cells were transfected with shRNAs together with the VSVG and gag-pol plasmids in a 3:1:1 ratio using the Fugene transfection reagent (Promega, Madison, WI, USA). Media containing viral particles was then collected 48 h after transfection, passed through a 0.22-μm filter, and added to C4-2 cells along with polybrene (10 μg/ml). After overnight incubation at 37°C, the media was replenished and cells were selected for puromycin resistance (2 μg/ml) for 3 days, after which knockdown was further validated. As controls pLKO.1 vector (SHC001, Sigma-Aldrich) and non-target shRNA-expressing stable cell lines were generated similarly.

### Ku70 DUB assay

HEK 293T cells were transiently transfected with His-Ub and Flag-KU70 at a ratio of 1 to 1.5. At 30 h post transfection, cells were irradiated. MG132 was added at 30 min before irradiation. Cells were then harvested 4 h post irradiation. Cells were lysed and sonicated in lysis buffer containing 30 mM HEPES, pH 7.4, 150 mM NaCl, 0.1% Tween 20, protease and phosphatase inhibitors (Thermo Scientific, Waltham, MA, USA), 10 mM NEM and DUB inhibitor. The denatured lysates were diluted with same buffer and immunoprecipitated with anti-Flag beads overnight. Bound Flag-KU70 was incubated either with recombinant USP14 (Boston Biochem-R&D Systems, Minneapolis, MN, USA) in DUB assay buffer (50 mM HEPES, pH 8.0, 50 mM NaCl, 1 mM EDTA, 10 mM DTT and 5% glycerol) at 30°C for 1.5 h for the DUB activity measurement. Samples were eluted with 2× Laemmli sample buffer and run on SDS-PAGE and immunoblotted against Flag (Cell-Signaling) and poly-Histidine monoclonal antibodies (Sigma), respectively.

### Immunoblotting and immunoprecipitation

Cells were lysed following the respective treatments in cell lysis buffer containing 50 mM Tris pH 7.4, 120 mM NaCl, 5 mM EDTA, 0.5% NP40 and 1 mM DTT, supplemented with the phosphatase inhibitor cocktail I, II (Sigma) and complete protease inhibitor tablet (Roche Diagnostics, Indianapolis, IN, USA). For immunoprecipitation, cell lysis was prepared in the above cell lysis buffer except that NP40 was reduced to 0.1%.

### Confocal immunostaining

Confocal immunostaining was done as described before (25). Briefly, cells were plated at 2 × 10^5^ cells/cm^2^ on 22 × 22-mm coverslips in 35-mm culture dishes. Immunostaining was performed as previously described (26). Briefly, following the respective treatments, cells were fixed with 2.0% paraformaldehyde in phosphate buffer saline (PBS) for 15 min at room temperature, washed 3× for 10 min each, permeabilized with 0.1% Triton X-100 in PBS for 10 min, and blocked in 10% fetal bovine serum in PBS for 1 h. The coverslips were then immunostained using the primary antibodies diluted in blocking buffer, followed by fluorescently-conjugated secondary antibody, washed 3× for 10 min each, and mounted using vectashield containing DAPI. Images were collected using an HCX Plan Apo 63×/1.4N.A. oil immersion objective lens on a Leica TCS-SP2 confocal microscope (Leica Microsystems AG, Buffalo Grove, IL, USA). Quantification was based on data observed from at least 50 cells.

### Chromatin recruitment assay

PCa cells were subjected to a chromatin recruitment assay as described (27). Briefly, cells were lysed with extraction buffer containing hydroxyethyl piperazineethanesulfonic acid (HEPES), ethylene diamine tetraacetic acid (EDTA), protease inhibitors, phosphatase inhibitors, and Triton X-100. Cells were incubated on ice for 20 min, and the pellet was collected after centrifuging at 14 000 g for 4 min. The pellet was incubated at room temperature in a shaker for 30 min after addition of the same extraction buffer, except that Triton X-100 was excluded and RNAse A was included in the buffer. The pellet was collected again after centrifugation at 14 000 g for 4 min. The pellet was suspended in PBS containing 1% SDS. The sample was heated for 10 min and sonicated for 10 s before separation by SDS-PAGE and immunoblotting with the indicated antibodies.

### Mass spectrometry identification of USP14-interacting proteins

C4-2 cells were transfected with Flag-HA-USP14 expression plasmid using Amaxa Cell Nucleofector Kit L (Lonza, Walkersville, MD, USA) and USP14 was immunoprecipitated using anti-Flag M2 magnetic beads (Sigma). The bound Flag fusion protein was eluted by competitive elution using 3× Flag peptide (Sigma). For the protein digestion, the samples were concentrated using a 3000 MW filter and solubilized in 50 μl 6 M urea buffer. The samples were reduced with DTT and alkylated with iodoacetamide. The urea concentration was diluted by adding 50 mM ammonium bicarbonate. An aliquot of trypsin, 5 μl 10 ng/μl trypsin in 50 mM ammonium bicarbonate, was added and incubated overnight at room temperature. A second aliquot of trypsin was added and digestion was continued for an additional 8 h. The digested samples were desalted using a PepClean C-18 spin column and the peptides were then resuspended in 1% acetic acid to make up a final volume of ∼30 μl for LC–MS analysis. The LC–MS system was a Finnigan LTQ-Obitrap Elite hybrid mass spectrometer system. The HPLC column was a Dionex 15 cm × 75 μm id Acclaim Pepmap C18, 2 μm, 100 Å reversed phase capillary chromatography column. Five microliter volumes of the extract were injected and the peptides eluted from the column by an acetonitrile/0.1% formic acid gradient at a flow rate of 0.25 μl/min were introduced into the source of the mass spectrometer on-line. The digests were analyzed by capillary column LC tandem MS and the CID spectra searched against the human UniProtKB database with the search program Mascot. All data was filtered based on a Mascot ion score >40. In addition, the data was searched specifically against the sequence of human USP14 using the program Sequest.

### Luciferase DNA repair reporter assay

A luciferase assay for NHEJ was used as described previously (28).

### Statistical analyses

Statistical comparisons between two groups were conducted by using the Student's *t* test and between multiple groups using two-way ANOVA using the Prism software.

## RESULTS

### Autophagy inhibition leads to increased USP14 IRIF formation

Autophagy is known to regulate genomic stability either indirectly such as via modulation of ROS (29,30) or directly through selective degradation of nuclear components (31,32) and/or targeting DNA double-strand break (DSB) repair-associated proteins (11,12). We recently identified USP14 as a critical link between autophagy and DSB repair signaling. We found that inhibition of autophagy upregulates USP14, which, in turn, negatively regulates RNF168, an E3 Ub ligase that is essential for 53BP1 recruitment at DNA DSBs (13). Since 53BP1 recruitment is critical for downstream activation of NHEJ (4), here we investigated the role of USP14 in regulating NHEJ repair in response to IR-induced DNA damage in autophagy-deficient cells.

**Figure 1.**
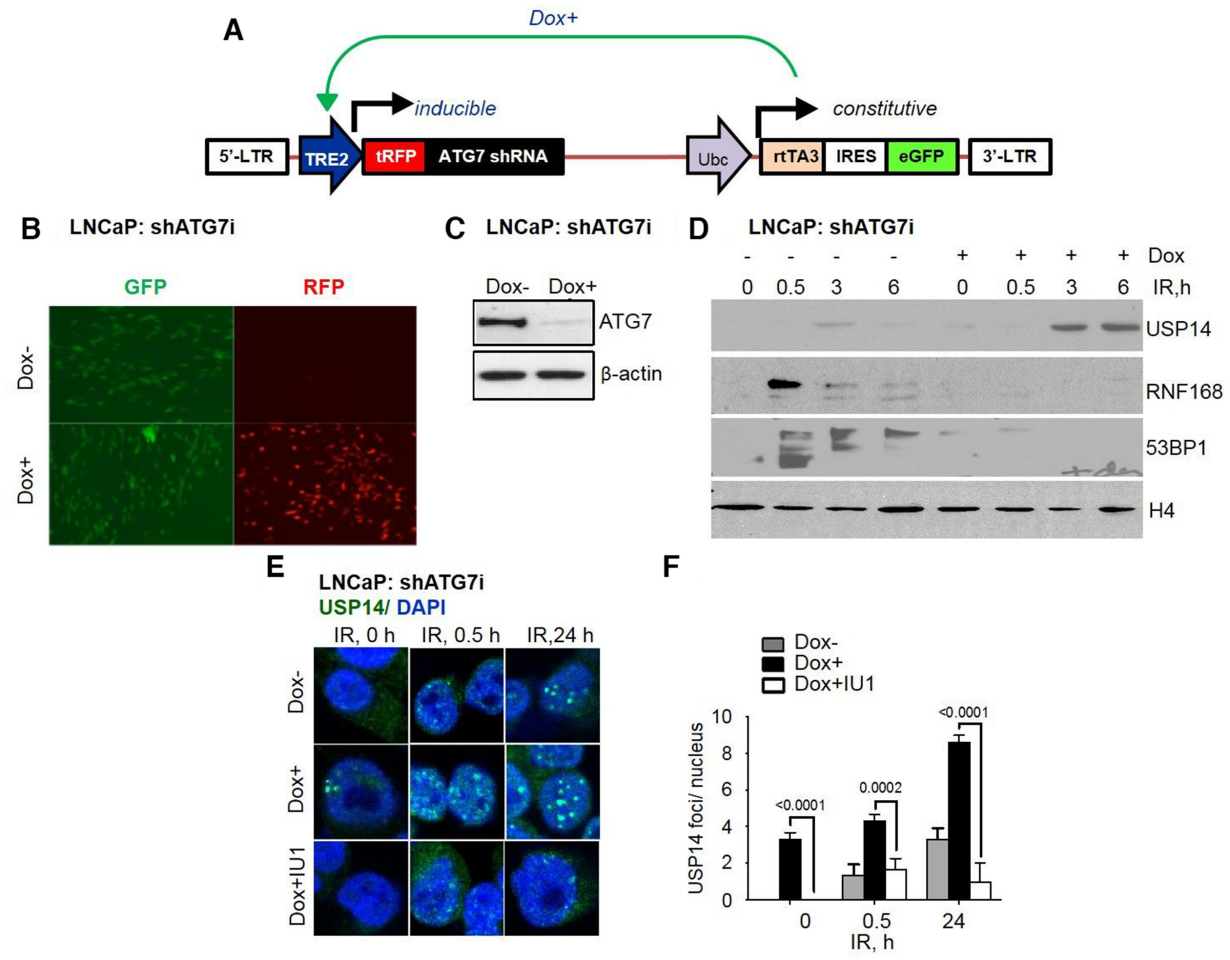
Increased USP14 chromatin recruitment in shATG7-inducible cells. (**A**) Diagram of pINDUCER-shATG7 system. pINDUCER-shATG7 encodes a constitutive cassette (rtTA3 and eGFP) and an inducible transcript (shATG7 and tRFP). (**B**) Fluorescence microscopy images of pINDUCER –shATG7-expressing (shATG7-inducible) LNCaP cells in the presence or absence of 800 ng/ml doxycycline (dox) for 72 h showing GFP and RFP expression. (**C**) Western blot analysis for ATG7 in shATG7-inducible LNCaP cells following indicated treatments. β-actin was used as the loading control. (**D**) Chromatin recruitment of USP14, RNF168, 53BP1, and H4 in shATG7-inducible LNCaP cells following dox ± 4 Gy IR. (**E**) Representative confocal images and (**F**) quantification of USP14 IRIFs at the indicated time points in shATG7-inducible LNCaP cells that were treated with dox for 72 h, and preincubated with 50 μM IU1 for 1 h prior to 4 Gy IR treatment. Nuclei were stained with DAPI. Statistical significance was determined using Student's *t*-test and two-way Anova (*n* = 3). *P* < 0.01*, *P* < 0.001**, *P* < 0.0001***.

To study the role of autophagy we used pharmacological inhibition by chloroquine (CQ) pretreatment (33), or a genetic approach with an inducible shRNA-mediated knockdown of ATG7 (autophagy related 7), using the pINDUCER system to inhibit autophagy (24). The lentiviral-based pINDUCER system constitutively encodes an rtTA3 and eGFP marker bicistronic transcript (Figure 1A and B). The addition of doxycycline (dox) induces transcription of the turboRFP (tRFP)-shRNA cassette (Figure 1A and B). LNCaP cells with pINDUCER-shATG7 (LNCaP:shATG7i) (24) were cultured in the absence (−dox) or presence (+dox) of 800 ng/ml dox for 72 h. Fluorescent microscopic analysis shows that in the absence of dox, <1.0% of cells are tRFP positive, demonstrating the tight regulation of pINDUCER (Figure 1A and B). Dox treatment led to tRFP fluorescence in >90% of cells, indicating that these cells express the tRFP-shATG7 cassette (Figure 1A and B). Expression of shATG7 is further confirmed by ATG7 protein depletion using immunoblotting analysis in the +dox compared to –dox cells (Figure 1C).

Next, we monitored the expression level of cytosolic LC3-I, an autophagy marker that is converted to the lipidated form, LC3-II, a gold standard marker of functional autophagy (33). We found that the LC3-II level was increased at 24 h following IR in −dox cells. In contrast, LC3-II was not expressed in +dox shATG7-expressing LNCaP cells (Supplementary Figure S1A). In addition, levels of sequestome1 (SQSTM/p62), a known substrate of autophagy, were higher at 24 h following IR in +dox cells suggesting higher accumulation of p62 due to inefficient clearance in autophagy-deficient cells (Supplementary Figure S1A). These data established that the shATG7-inducible LNCaP cells were indeed deficient in autophagy induction in response to IR.

Next, to study the role of USP14 in regulating DNA DSB response in autophagy-deficient cells, we used biochemical fractionation to investigate USP14 recruitment to DSBs. Inhibition of autophagy resulted in higher basal and IR-induced chromatin-bound USP14 in dox+ LNCaP (Figure 1D) and CQ pretreated C4-2 cells (Supplementary Figure S1B). Consistent with the role of USP14 in regulating chromatin recruitment of RNF168 and 53BP1, an increase in USP14 levels resulting from inhibition of autophagy led to reduced IR-induced chromatin- bound RNF168 and 53BP1, thus suggesting impaired NHEJ signaling in these cells (Figure 1D and Supplementary Figure S1B).

IR-treatment alone induced USP14 IRIF foci formation in LNCaP cells in a time-dependent manner, similar to what we have previously shown in C4-2 and PC-3 cells (13). Strikingly, the number of USP14 foci were increased in shATG7-expressing LNCaP cells at all time points following IR treatment. Importantly, USP14 IRIF formation was significantly (*P* < 0.0002) diminished in shATG7-expressing cells pretreated with the USP14 inhibitor IU1 before IR treatment (Figure 1E and F), suggesting that the catalytic activity of USP14 was needed for its recruitment to DSB sites.

### USP14 regulates NHEJ-associated DDR signaling

**Figure 2.**
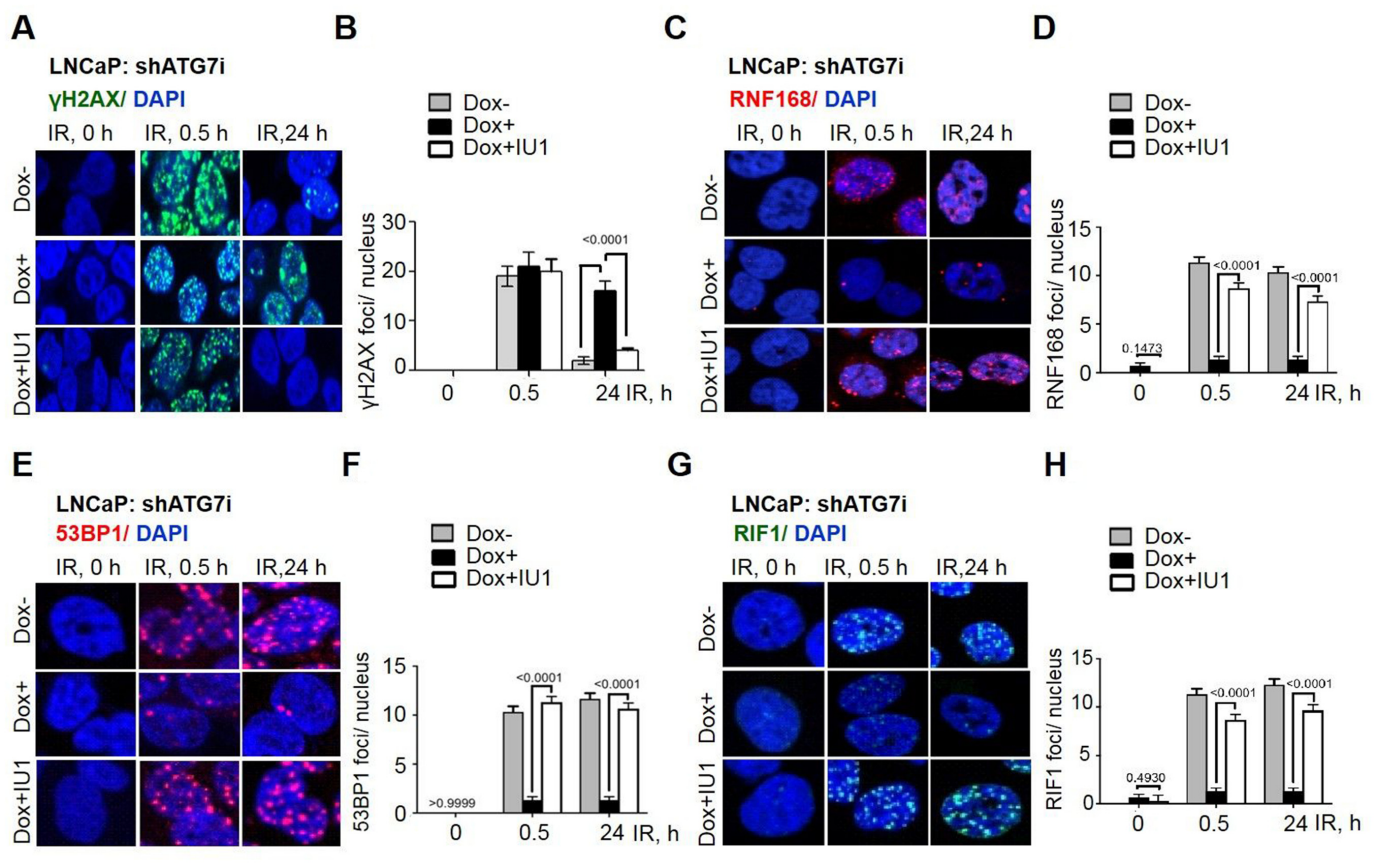
USP14 inhibits DDR signaling in shATG7-inducible cells. Confocal immunostaining and graphical representation of IRIFs for (**A**, **B**) γ-H2AX, (**C**, **D**) RNF168, (**E**, **F**) 53BP1, and (**G**, **H**) RIF1 at the indicated time points in shATG7-inducible LNCaP cells that were treated with dox for 72 h, and preincubated with 50 μM IU1 for 1 h prior to 4 Gy IR treatment. Nuclei were stained with DAPI. Data shown are the means ± SEM (*n*  =  2); *P* < 0.05 *, *P* < 0.01**.

When DSBs are detected, a signaling cascade is initiated by the ATM-mediated phosphorylation of H2AX at Ser-139 (γH2AX) that leads to downstream recruitment of the essential DDR proteins, including RNF8, RNF168 and 53BP1 (4). So, next, we studied DDR signaling in response to IR in dox-inducible shATG7 expressing LNCAP cells. γH2AX foci formation, a gold standard marker for DSBs (34) occurred in response to IR in both dox+ and dox− cells at 0.5 h (Figure 2A and B), indicating similar induction of DSBs. However, substantially reduced γH2AX foci were observed only in dox- cells and not the shATG7-expressing cells at a later time point, *i.e*. at 24 h (Figure 2A and B), indicating that the DSB repair is impaired in autophagy-deficient cells. Also, formation of RNF168 (Figure 2C and D) and 53BP1 (Figure 2E and F) IRIFs were greatly diminished in autophagy-deficient (dox+) cells. Strikingly, pharmacological inhibition of USP14 using IU1 could significantly (*P* < 0.0001) restore DSB repair as determined by resolution of γH2AX foci (Figure 2A and B), and restored RNF168 and 53BP1 (Figure 2C–F) IRIFs in dox+ cells. Overall, these data indicate that acute, inducible shATG7-expressing cells regulate DDR signaling by USP14 as do cells with chronic, stable shATG7 expression (13).

RIF-1 is an anti-DNA resection factor that is recruited to DSB sites downstream of phosphorylated 53BP1 that acts to antagonize DNA resection that leads to HR and thus promote NHEJ (35). Thus, we assessed whether RIF1 was associated with the 53BP1 foci formation in IU1-treated autophagy-deficient cells. Indeed, in autophagy-deficient cells the number of RIF1 IRIFs were reduced (Figure 2G and H) and could be significantly (*P* < 0.0001) restored to the levels found in untreated cells following IU1 treatment in dox+ cells (Figure 2G and H). Overall, these findings indicate that USP14 negatively regulates DDR signaling associated with NHEJ in response to IR in autophagy-deficient cells.

### USP14 prevents chromatin recruitment of core NHEJ proteins

**Figure 3.**
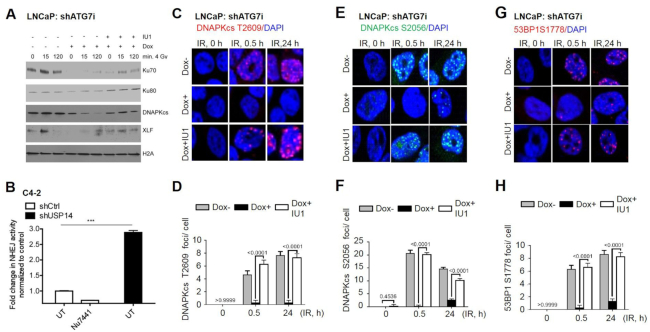
USP14 disrupts NHEJ DDR in autophagy-deficient cells. (**A**) Chromatin recruitment of the indicated proteins in shATG7-inducible LNCaP cells following +/- dox +/- IU1 +/- 4Gy IR at the indicated time points. H4 was used as loading control for the chromatin-bound fraction. (**B**) NHEJ measured by a plasmid luciferase repair assay in shCtrl- versus shUSP14-expressing C4-2 cells. Nu7441 treatment in shCtrl-expressing C4-2 cells was used as a negative control. Data are normalized for transfection efficiency and then to uncut luciferase. Confocal immunostaining and graphical representation of quantitation of (**C, D**) DNA-PKCs-T2609, (**E, F**) DNA-PKCs-S2056, (**G, H**) 53BP1-S1778 IRIFs at the indicated time points in shATG7-inducible LNCaP cells following ± dox ± IU1 ±± 4Gy IR. Nuclei were stained with DAPI. Data shown are the means ± SEM (*n*  =  2); *P* < 0.05 *, *P* < 0.01**.

ATM mediates phosphorylation of the N-terminal domain of 53BP1, which is required for the recruitment of the anti-resection factors RIF1 and PTIP to the DSBs (18,19). 53BP1 then inhibits HR and promotes NHEJ, initiated by binding of the Ku70/Ku80 heterodimer to two ends of the DSB, followed by recruitment of DNA-PKcs. DNA-PKcs undergoes autophosphorylation at T2609 and S2056 and subsequently phosphorylate other NHEJ component proteins, including 53BP1 at S1778. Ultimately, the DNA ligase IV/XRCC4/XLF complex ligates the DNA ends (2,36–38). We, therefore, investigated the role of USP14 in regulating recruitment of the NHEJ core complex in response to IR by chromatin extraction. The core NHEJ-complex proteins, including Ku70, Ku80, DNA-PKcs, and XLF were recruited to chromatin in autophagy-proficient cells in response to IR treatment (Figure 3A). However, chromatin-recruitment of NHEJ core factors was dramatically reduced in response to IR in autophagy-deficient cells (Figure 3A). Interestingly, in IU1-treated cells the recruitment of Ku70, Ku80, DNA-PKcs and XLF was efficiently restored in autophagy-deficient cells (Figure 3A) The NHEJ-complex assembly at the DNA DSBs is essential for efficient NHEJ repair (2). So, next we examined whether USP14 regulates NHEJ DNA repair using a well-established host-cell reactivation reporter assay (28). We found that shUSP14- expressing C4-2 cells had significantly increased levels of NHEJ compared to shCtrl-expressing cells (Figure 3B). Collectively, these data show that USP14 indeed regulates NHEJ-complex assembly in autophagy-deficient cells.

To further assess the role of USP14 in NHEJ repair, we examined the status of DNA-PKcs autophosphorylation on S2056 and T2609, and of a DNA-PKcs substrate, 53BP1 on S1778. Indeed, the number of DNA-PKcs S2056, T2609 and 53BP1 S1778 (Figure 3C–H) IRIFs were greatly reduced at 0.5 and 24 h in autophagy-deficient cells. However, in IU1-treated autophagy-deficient cells, DNA-PKcs autophosphorylation and 53BP1 S1778 phosphorylation-dependent IRIFs were significantly (*P* < 0.0001) restored in shATG7-expressing LNCaP cells (Figure 3C–H). Similarly, inhibition of autophagy by CQ pretreatment in C4-2 cells resulted in reduced DNA-PKcs S2056, T2609 and 53BP1 S1778 (Supplementary Figure S2A–F) IRIFs following IR, indicating that the NHEJ repair is impaired upon inhibition of autophagy. Importantly, USP14 inhibition by IU1 treatment effectively restored NHEJ repair signaling. Overall, these data provide substantial evidence that USP14 is an important regulator of DDR and NHEJ repair signaling.

### AKT regulates the function of USP14 as a DDR effector

**Figure 4.**
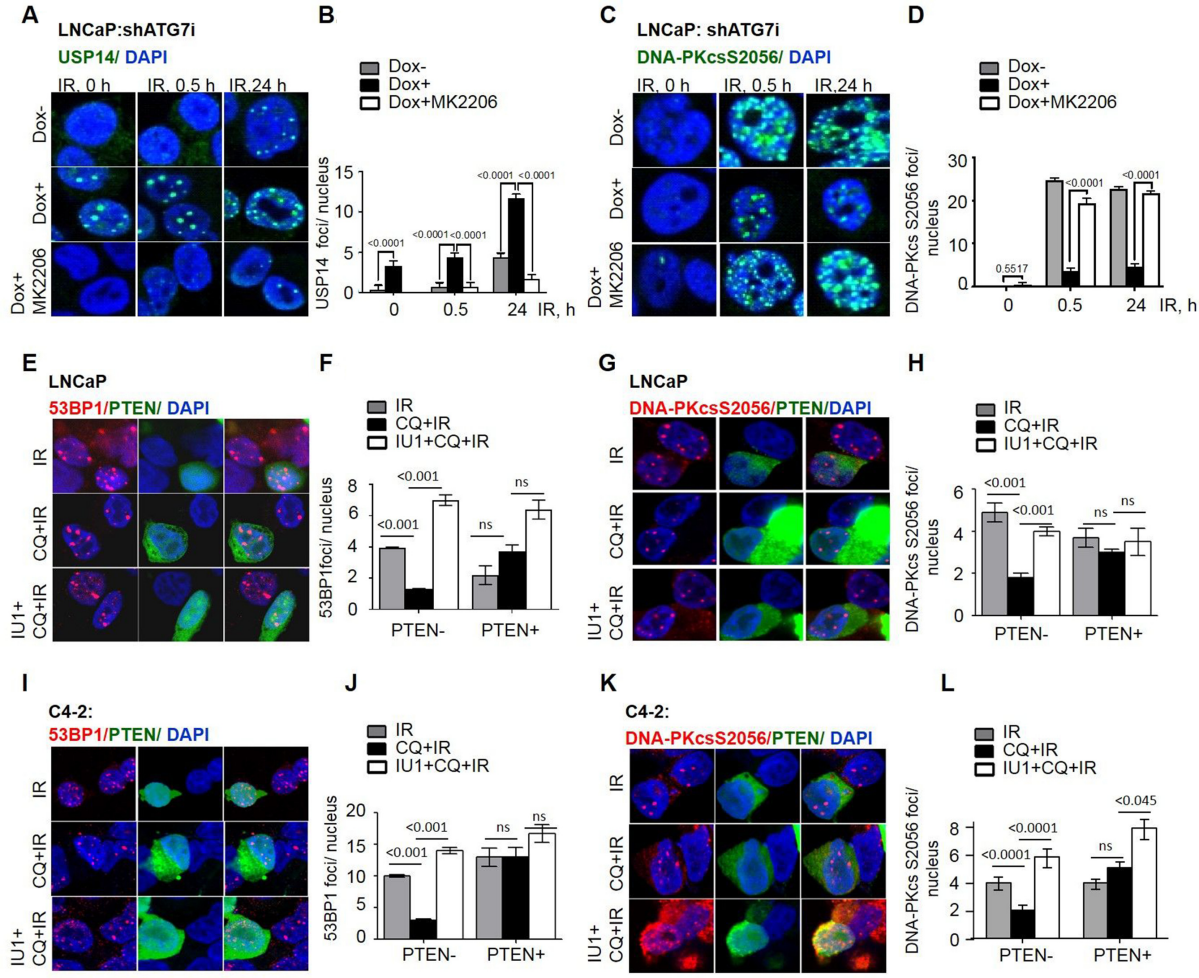
Constitutive Akt regulates USP14 IRIF and NHEJ DDR signaling in autophagy-deficient cells. Confocal immunostaining and graphical representation of (**A**, **B**) USP14, (**C**, **D**) DNA-PKCs-S2056IRIF at the indicated time points in shATG7-inducible LNCaP cells that were treated with dox and preincubated with 10 μM MK2206 for 1 h prior to the 4Gy IR treatment. LNCaP cells were transfected with GFP-PTEN, (**E**, **F**) 53BP1 and (**G**, **H**) DNA-PKcs S2056 IRIF formation were analyzed in PTEN+ *vs*. PTEN- cells, following indicated treatments. C4-2 cells were transfected with GFP-PTEN, and (**I**, **J**) 53BP1 and (**K**, **L**) DNA-PKcs S2056 IRIF formation were analyzed in PTEN+ versus PTEN- cells, following indicated treatments. Nuclei were stained with DAPI. Data shown are the means ± SEM (*n*  =  2); *P* < 0.05 *, *P* < 0.01 **.

As the proteasomal activity of USP14 is regulated by Akt-dependent phosphorylation of USP14 on S432 (23), we investigated whether AKT impacts on the nuclear function of USP14. We first examined whether AKT regulates IRIF formation by USP14. As shown above, we found that IR-treatment induced USP14 foci formation in a time-dependent manner. Moreover, the number of USP14 foci was significanly (*P* < 0.0001) increased in shATG7-expressing LNCaP cells at all time points following IR treatment. Strikingly, an allosteric pharmacologic inhibitor of AKT, MK2206, significantly (*P* < 0.0001) reduced USP14 IRIFs in shATG7-expressing cells (Figure 4A and B). Moreover, DNA-PKcs S2056 (Figure 4C and D), DNA-PKcs T2609, 53BP1 S1778 and RIF1 foci (Supplementary Figure S3A–F) were dramatically reduced at 0.5 and 24 h following IR in autophagy-deficient cells. Strikingly, the DNA-PKcs autophosphorylation, 53BP1 S1778 phosphorylation, and RIF1 foci were significantly (*P* < 0.0001) restored in MK2206-treated autophagy-deficient cells (Figure 4C, D and Supplementary Figure S3A–F). Overall, these data indicate that Akt regulates the nuclear function of USP14 as a DDR effector.

To explore the role of PTEN in AKT-mediated USP14 phosphorylation in NHEJ repair, we examined IRIF by USP14 in VCaP (PTEN+) versus LnCaP (PTEN-) cells following IR. VCaP cells that were indeed PTEN+ and as they expressed inactive AKT as shown by absence of pAKT(Supplementary Figure S4A), failed to form nuclear USP14 IRIF (Supplementary Figure S4B and C). Remarkably, CellMiner database analysis found a strong positive correlation between mRNA expression of USP14 and AKT1 in PCa cell lines (Supplementary Figure S4D). To directly examine the role of PTEN, we generated isogenic paired cell systems with and without ectopic expression of GFP-PTEN in two PTEN-deficient PCa cell lines. PTEN overexpression led to reduced pAKT in both LNCaP (Supplementary Figure S4E) and C4-2 (Supplementary Figure S4F) cells. USP14 IRIFs were formed in response to IR treatment in both (PTEN–) LNCaP (Supplementary Figure S4G and H) and C4-2 cells (Supplementary Figure S4I and J). In contrast, LNCaP and -C4-2 cells with ectopic expression of GFP-PTEN (PTEN+) showed significantly reduced USP14 foci formation in response to IR (Supplementary Figure S4G–J).

Moreover, as expected, IR-induced 53BP1 (Figure 4E, F, I and J), and DNA-PKcs S2056 (Figure 4G, H, K and L) foci formation that were reduced in (PTEN–) LNCaP and C4-2 autophagy-deficient cells, were significantly (*P* < 0.0001) restored in IU1-treated autophagy-deficient cells (Figure 4E–L). In contrast, in (PTEN+) LnCaP and C4-2 cells neither autophagy deficiency nor USP14 deficiencies had any remarkable effect on IR-induced 53BP1 or DNA-PKcs S2056 foci formation (Figure 4E–L).

Taken together, these data establish that elevated AKT signalling plays a critical role in regulating the function of USP14 in DSB repair in PTEN- and autophagy-deficient cancer cells.

### USP14 directly interacts with Ku70 and targets its ubiquitination

**Figure 5.**
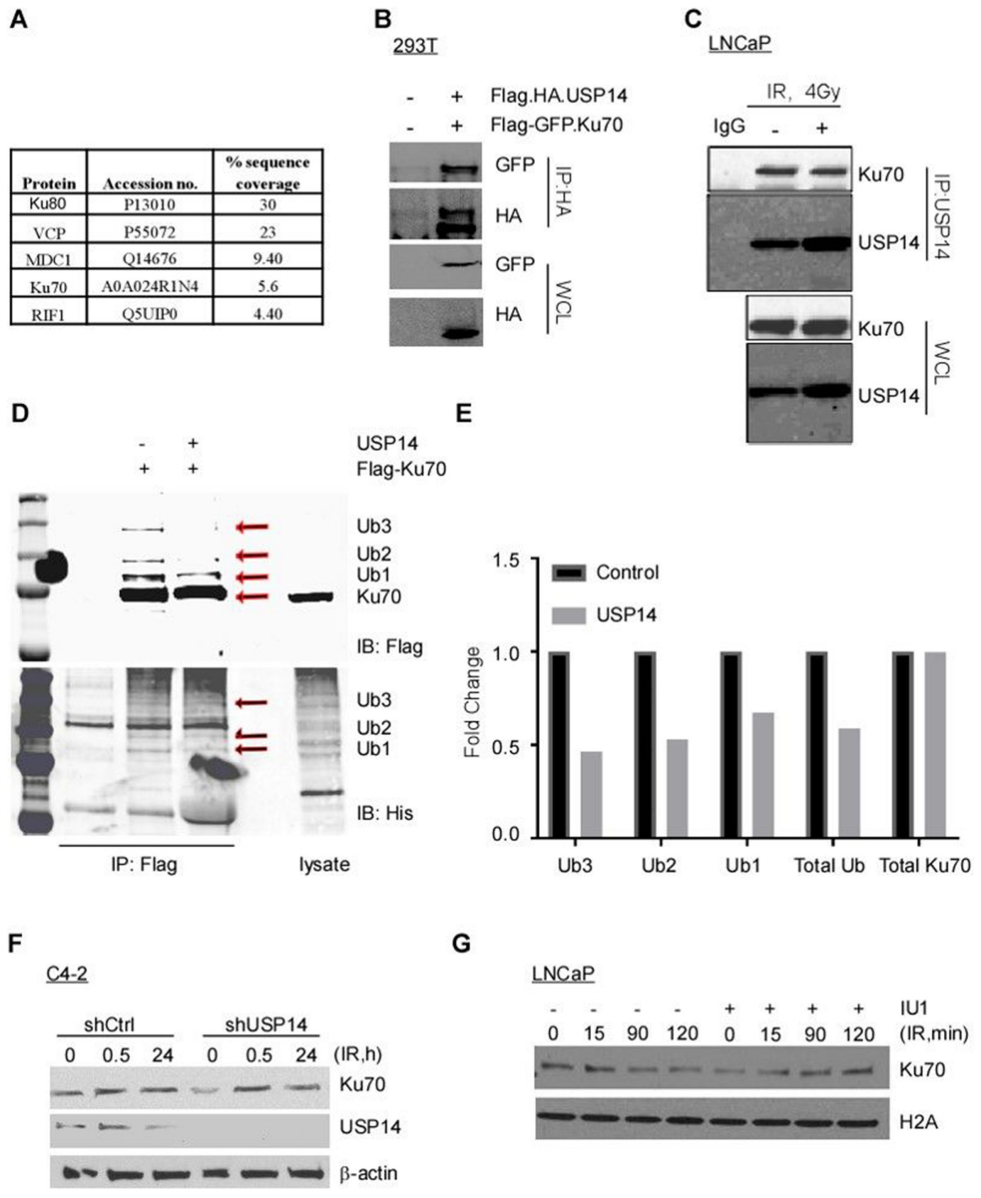
USP14 directly interacts with Ku70 and targets its ubiquitination. (**A**) List of NHEJ associated proteins that were found to interact with USP14 using Mass spectrometric analysis. (**B**) HEK 293T cells were transfected with the indicated constructs, followed by immunoprecipitation using anti-HA antibody and immunoblotting using the indicated antibodies. The corresponding whole cell lysates (WCLs) were used as input controls and probed with the indicated antibodies. (**C**) LNCaP cells were treated with 4 Gy IR, as indicated, and USP14 was immunoprecipitated followed by immunoblotting against indicated antibodies. (**D**) HEK 293T cells were transfected with His-Ub and Flag-KU70, followed by MG132 and 10 Gy IR treatment. Cells were lysed under denaturing conditions, followed by immunoprecipitation of Flag-KU70 and incubation either with or without recombinant USP14. Eluted samples were separated on SDS-PAGE and immunoblotted as indicated. (**E**) Quantitative representation of Ku70-Ub determined in D. (**F**) Western blot analysis for Ku70 and USP14 at the indicated times following 4 Gy IR treatment in C4-2 cells stably expressing shCtrl or shUSP14, β-actin was used as the loading control. (**G**) Chromatin recruitment of Ku70 in LNCaP cells following IU1 ± 4Gy IR treatments at the indicated time points. H2A was used as loading control for the chromatin-bound fractions.

To obtain further insights into USP14 function and regulation, we aimed to identify USP14-interacting proteins by mass spectrometry. FLAG-HA-tagged USP14 was transiently expressed and purified from C4-2 cells and subjected to mass spectrometry analysis. Along with a number of known proteasome-associated proteins that were co-purified with USP14, including PMSB1, PMSA2, and PSMD4 (Supplementary Table 1), we also identified several core NHEJ proteins (Figure 5A) among USP14-interacting proteins.

We chose to pursue the USP14 interaction with Ku70 and Ku80 because ubiquitination is a critical post-translational modification for Ku70/80 dissociation from chromatin post NHEJ for efficient ligation of broken DNA ends (39–42). We first confirmed the interaction between Ku70 and USP14 by performing immunoprecipitation (IP) of USP14 from HEK 293T cells co-expressing Flag-HA-USP14 and EGFP-Flag-Ku70 using anti-HA, which co-immunoprecipitated USP14 and Ku70 (Figure 5B). We further confirmed the interaction between endogenous Ku70 and USP14 by IP of USP14 from LNCaP cells followed by immunoblotting for Ku70 (Figure 5C). We next asked if Ku70 was a direct target of USP14 for deubiquitination by using poly-ubiquitinated Ku70 as the substrate in an *in vitro* deubiquitination assay. HEK 293T cells transfected with His-Ub and Flag-Ku70 were treated with IR. The poly-ubiquitinated Ku70 form was purified using anti-Flag beads and incubated with recombinant human USP14 for the *in vitro* deubiquitinating assay. We observed that ubiquitination of Ku70 was decreased in the presence of USP14 (Figure 5D and E). However, while we did observe an interaction between USP14 and Ku80, USP14 showed no effect on ubiquitination of Ku80 in our *in vitro* assay (data not shown). Thus, these data indicate that Ku70 but not Ku80 is a direct substrate of USP14 for deubiquitination.

Ubiquitination of proteins typically regulates their protein stability. Moreover, ubiquitination is also known to regulate Ku70/80 dissociation from chromatin following completion of NHEJ (39–42). Therefore, we examined whether USP14 deubiquitinates Ku70 and regulates its protein stability and/ or its chromatin association in response to IR-induced DNA DSBs. Comparison of Ku70 levels at different time points following IR treatment showed no change in shUSP14- *vs* shCtrl-expressing C4-2 cells (Figure 5F). However, chromatin recruitment of Ku70 at different time points following IR treatment increased following inhibition of USP14 by IU1 (Figure 5G). These results suggest that USP14 could regulate the chromatin recruitment of the Ku70/80 complex without notably affecting expression of these component protein levels.

Overall, these findings establish USP14 as a critical regulator of NHEJ DSB repair pathway in autophagy and PTEN-deficient PCa cells. USP14 antagonizes 53BP1 recruitment and also directly targets Ku70-ubiquitination which, in turn, inhibits NHEJ core assembly at DSB sites in autophagy-deficient PCa cells. USP14 inhibition or knockdown restored NHEJ core complex assembly and DSB repair signaling in PTEN- and autophagy-deficient cells.

## DISCUSSION

Here, we show that Ku70 is a direct target of USP14 for deubiquitination and that USP14 is a prominent determinant of regulation of NHEJ repair pathway by autophagy in PTEN-deficient PCa cell lines. Moreover, the DSB repair function of USP14 is dependent on Akt-mediated phosphorylation and activation of USP14. USP14 regulates NHEJ repair pathway by acting at two distinct levels. We have previously shown that USP14 inhibits RNF168-dependent ubiquitination signaling and 53BP1 recruitment to DSB sites (13). Here, we demonstrate that USP14 directly interacts with Ku70, and USP14-mediated deubiquitination regulates DSB- recruitment of Ku70 and downstream NHEJ-core complex assembly. As autophagy inhibition increases USP14 levels that accumulate on chromatin, it prevents DSB recruitment of Ku70 and downstream NHEJ-core complex assembly. Strikingly, USP14 inhibition or knockdown fully restored NHEJ core complex assembly and DSB repair signaling in autophagy-deficient cells.

Autophagy is important for maintenance of genomic stability through indirect mechanisms, such as regulation of ROS (29,30) or maintenance of nuclear integrity (31,32). While the role of autophagy in maintaining HR has been previously examined (11) (12), to our knowledge we are the first to identify a direct role of autophagy in regulating the NHEJ pathway. Thus, functional autophagy promotes NHEJ repair by regulating USP14, an inhibitor of the NHEJ repair.

The role of Ku70 in NHEJ is well established (2). The Ku70/80 heterodimer senses and binds DSBs, and this is central to initiation of recruitment of other components essential for NHEJ repair. Following the completion of DNA repair, the Ku70/80 heterodimer is removed from the DSB ends in a ubiquitination-dependent manner. Previously, NEDD8-dependent ubiquitin ligases RNF8, RNF138, and RNF126 have been implicated in ubiquitylation of Ku70/80 (39–42). Here, we describe for the first time deubiquitination of Ku70 by USP14 as an important mechanism that regulates chromatin recruitment of Ku70/80 and subsequently downstream NHEJ core assembly at DSB sites. While ubiquitination has been shown to be essential for removal of Ku70/80 in previous studies (39–42), we find that deubiquitination of Ku70 by USP14 results in reduced Ku70 recruitment to DSB sites. Whether the early stage ubiquitination that is required for chromatin recruitment of Ku70 involves one or more previously known E3 ligases, and which lysine residues of Ku70 does it involve still needs to be investigated in future studies.

Loss of PTEN function significantly correlates with poor survival, resistance to chemotherapy, and advanced disease in many human cancers, including prostate (43). We identify AKT-mediated phosphorylation of USP14 as a negative regulator of NHEJ-DDR in autophagy-deficient cells. Inactivation of PTEN leading to constitutively active AKT-signaling has been shown to directly regulate DSB repair in various studies (44,45). AKT has been shown to promote NHEJ through interaction with DNA-PKcs that leads to its recruitment to DNA DSBs and its removal at later stages (46,47). Conversely, an inhibitory role of AKT in NHEJ was also reported. Thus, phosphorylation of XLF by AKT resulted in its dissociation from the XRCC4/DNA ligase IV complex, and subsequently its cytoplasmic translocation followed by ubiquitination and degradation of XLF (48). The discrepancies in the published reports on the role of PTEN/AKT in DSB repair can be attributed to the complex interactions between PTEN/AKT and DDR at various levels in different cellular context. Complete PTEN loss occurs only during late stages of PCa (49), thus, alternative genomic adaptations to tumor progression may regulate DSB repair in a PTEN/AKT-independent manner. Clearly, we found that PTEN-positive PCa cells that lack constitutive AKT activation failed to form USP14 foci in response to IR. And, despite inactive AKT/USP14 VCaP cells demonstrated an NHEJ-defective phenotype as we reported earlier (27).

Our data establish that autophagy is critical for determining the effect of PTEN in regulating NHEJ through USP14. Importantly while the disruption of AKT, either by pharmacological inhibition or PTEN overexpression, restored NHEJ-associated DDR signaling defects mediated by USP14 in autophagy-deficient PTEN-null cells, PTEN overexpression alone had no considerable effect on NHEJ signaling despite also decreasing USP14 foci. This suggests that in autophagy-proficient PCa cells alternative PTEN/USP14-independent mechanisms regulate NHEJ, and PTEN-dependent USP14 regulation alone may not be sufficient to show a robust effect on NHEJ repair. Clearly, in autophagy-deficient cells the PTEN/AKT/USP14 axis is a prominent factor regulating NHEJ.

We identify two important tumor-associated pathways, i.e. autophagy and PTEN/AKT to directly regulate the function of USP14 in DSB repair. In cells with constitutive AKT activation, inhibition of autophagy causes excessive DSB recruitment of USP14 that dampens DDR signaling and NHEJ. Physiologically, functional autophagy in healthy cells must maintain low levels of USP14 that results in balanced NHEJ that might otherwise lead to genomic rearrangements, such as DNA translocations, leading to carcinogenesis. Activation of carcinogenic signals such as: (i) loss of PTEN that leads to constitutive AKT activation that promotes USP14 function and (ii) mTORC1 activation that increases USP14 expression by inhibiting autophagy (13) inhibit NHEJ and promote tumorigenesis. Indeed, overexpression of USP14 is associated with poor survival in a variety of cancers (19,20,50,51).

Disruption of USP14 in autophagy-deficient cells restores DDR signaling and DSB recruitment of NHEJ-associated factors in response to IR. Consistently, we have previously reported that increased radiosensitization of PCa cells upon inhibition of autophagy could be reversed by inhibition of USP14 (13). These findings have important therapeutic implications. First, autophagy signaling is upregulated in advanced cancers, including high-grade PCa (52,53). Moreover, aberrant constitutive activation of PI3K/AKT signaling has been identified in many cancers, including ∼40% of early PCa and 70–100% in advanced PCa (49). Therefore, by elucidating the mechanism underlying regulation of NHEJ by USP14, our study identifies inhibition of autophagy as a potential IR-sensitizing mechanism based on its ability to upregulate USP14 in cells with constitutive activation of AKT. These studies lay a foundation for defining the radiosensitizing effect of autophagy inhibition in patients with constitutive activation of AKT signaling, based on the AKT/USP14 axis. Moreover, our data also imply that high USP14 levels can predict radiosensitivity in cancer patients.

## Supplementary Material

gkz1103_Supplemental_Files
