# Supplementary material for: USP14 is a deubiquitinase for Ku70 and critical determinant of non-homologous end joining repair in autophagy and PTEN-deficient cells
Source: Nucleic Acids Res. 2019 Nov 19;48(2):736–47. doi: 10.1093/nar/gkz1103 (PMC7145659; doi:10.1093/nar/gkz1103)
Supplement: gkz1103_Supplemental_Files [file gkz1103_supplemental_files.zip › Supplementary Figure Legends.docx]

**Supplementary Figure Legends**

**Supplementary Figure S1. Increased USP14 chromatin recruitment in autophagy-deficient cells. A**) Western blot analysis for LC3 and p62 following 4Gy IR treatment in shATG7-inducible LNCaP cells in the presence or absence of dox for 72 h. β-actin was used as the loading control. (**B**) Chromatin recruitment of USP14, RNF168, 53BP1, and H4 in C4-2 cells following CQ+/- 4Gy IR.

**Supplementary Figure S2. USP14 inhibits DDR signaling in autophagy-deficient PCa cells**. Confocal immunostaining and graphical representation of quantitation of (A, B) DNA-PKCs-T2609, (**C, D**) DNA-PKCs-S2056, (**E, F**) 53BP1-S1778, IRIF at the indicated time points in C4-2 cells CQ and preincubated with 50 μM IU1 for 1 h prior to the 4Gy IR treatment. Nuclei were stained with DAPI. Data shown are the means ± SEM (n = 2).

**Supplementary Figure S3. Constitutive Akt regulates NHEJ DDR signaling in autophagy-deficient LNCaP cells.** Confocal immunostaining and graphical representation of (**A, B**) DNA-PKCs-T2609, (**C, D**) 53BP1-S1778, and (**E, F**) RIF1 IRIF at the indicated time points in shATG7-inducible LNCaP cells that were treated with dox and preincubated with 10 μM MK2206 for 1 h prior to the 4Gy IR treatment. Nuclei were stained with DAPI. Data shown are the means ± SEM (n = 2).

**Supplementary Figure S4. Constitutive Akt regulates USP14 IRIF formation in PCa cell lines.** (**A**) Western blot analysis of PTEN, pAKT, and AKT in LNCaP and VCaP cells following 4Gy IR treatment at the indicated time points. (**B, C**) USP14 IRIFs at the indicated time points in LNCaP and VCaP cells. (**D**) Co-expression analysis using CellMiner of expression (exp, Z score microarray log2 intensity data) of AKT *vs* USP14 in PCa CCLE cell lines. Nuclei were stained with DAPI. Data shown are the means ± SEM (n = 2); P < 0.05 *, P < 0.01 **. Western blot analysis of pAKT and AKT in (**E**) LNCaP and (**F**) C4-2 cells +/- GFP-PTEN. USP14 IRIF formation in PTEN+ vs. PTEN- (**G,H**) LNCaP and (**I,J**) C4-2 cells, following the indicated treatments. Nuclei were stained with DAPI. Data shown are the means ± SEM (n = 2).

**Supplementary Table 1.** List of proteins interacting with USP14 identified through MS analysis.
