# Supplementary material for: USP14 is a deubiquitinase for Ku70 and critical determinant of non-homologous end joining repair in autophagy and PTEN-deficient cells
Source: Nucleic Acids Res. 2019 Nov 19;48(2):736–47. doi: 10.1093/nar/gkz1103 (PMC7145659; doi:10.1093/nar/gkz1103)
Supplement: gkz1103_Supplemental_Files [file gkz1103_supplemental_files.zip › Supplementary Table.docx]

Supplementary Table 1: USP14-interacting proteins identified by LC-MS/MS analysis

| **Protein** | **Accession number** | **% seq coverage** |
| --- | --- | --- |
| HNRNPL | P14866 | 57 |
| TXN | P10599 | 49 |
| UBE2N | P61088 | 44 |
| USP14 | P54578 | 43 |
| Proteasome subunit β type | Q86U62 | 35 |
| PSMD14 | O00487 | 22 |
| PSMD10 | B1AJY7 | 22 |
| PSMD7 | H3BTM8 | 21 |
| PSMD4 | P55036 | 19 |
| PSMC3 | P17980 | 18 |
| PSMA6 | P60900 | 23 |
| PSMB1 | P20618 | 22 |
| ECHS1 | P30084 | 41 |
| PSMC4 | P43686 | 13 |
| PSMD3 | O43242 | 13 |
| PSMD13 | A0A024R201 | 12 |
| PSMD11 | O00231 | 11 |
| PSMC1 | P62191 | 10 |
| PSMB5 | P28074 | 10 |
| USP15 | Q9Y4E8 | 8.6 |
| PSMD2 | Q13200 | 8 |
| PSMC6 | A0A087X2I1 | 5.7 |
| XPO1 | O14980 | 4 |
| PSMD1 | A0A087WW66 | 2.2 |

Note: All identified interacting proteins listed above were also confirmed in BioGrid^3.5^ (<https://thebiogrid.org/114551/summary/homo-sapiens/usp14.html>)
