# Supplementary figures and images for: USP14 is a deubiquitinase for Ku70 and critical determinant of non-homologous end joining repair in autophagy and PTEN-deficient cells
Source: Nucleic Acids Res. 2019 Nov 19;48(2):736–47. doi: 10.1093/nar/gkz1103 (PMC7145659; doi:10.1093/nar/gkz1103)

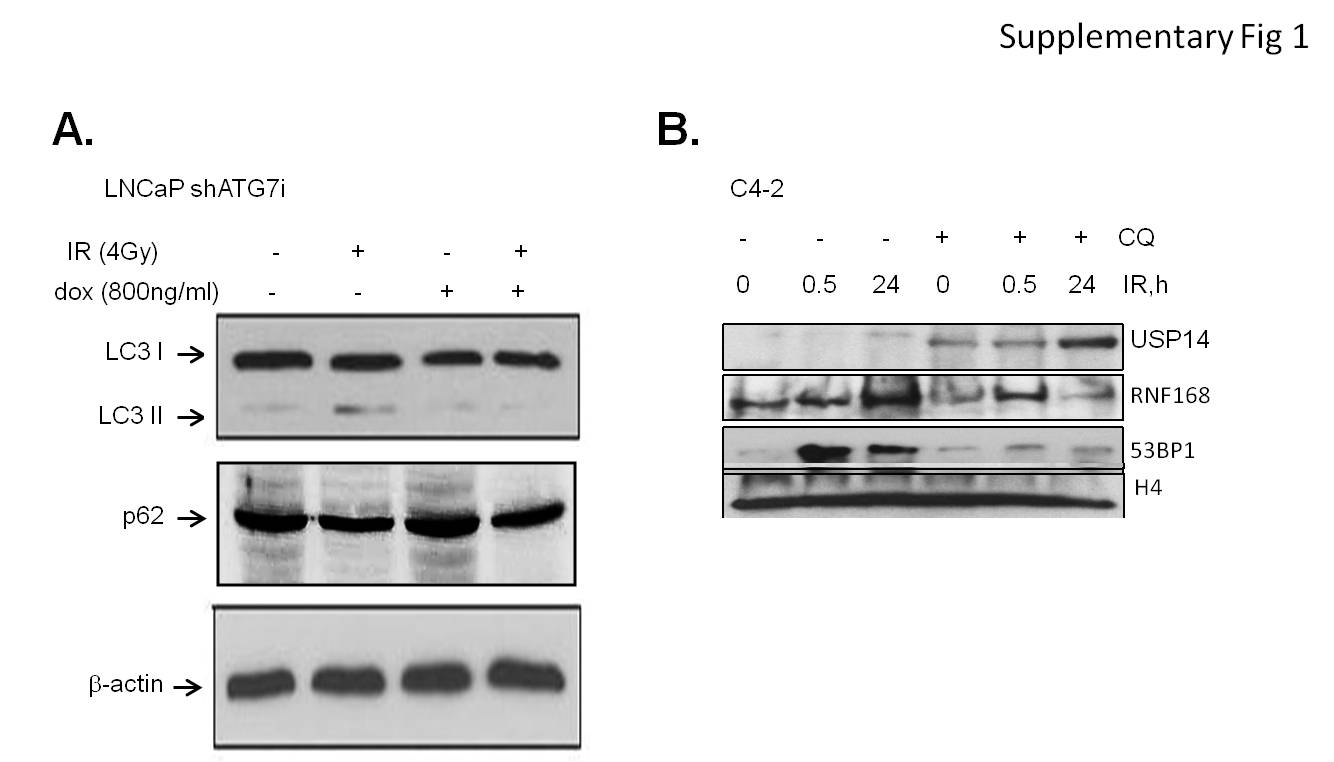

Supplement: gkz1103_Supplemental_Files [file gkz1103_supplemental_files.zip › S1.jpg]

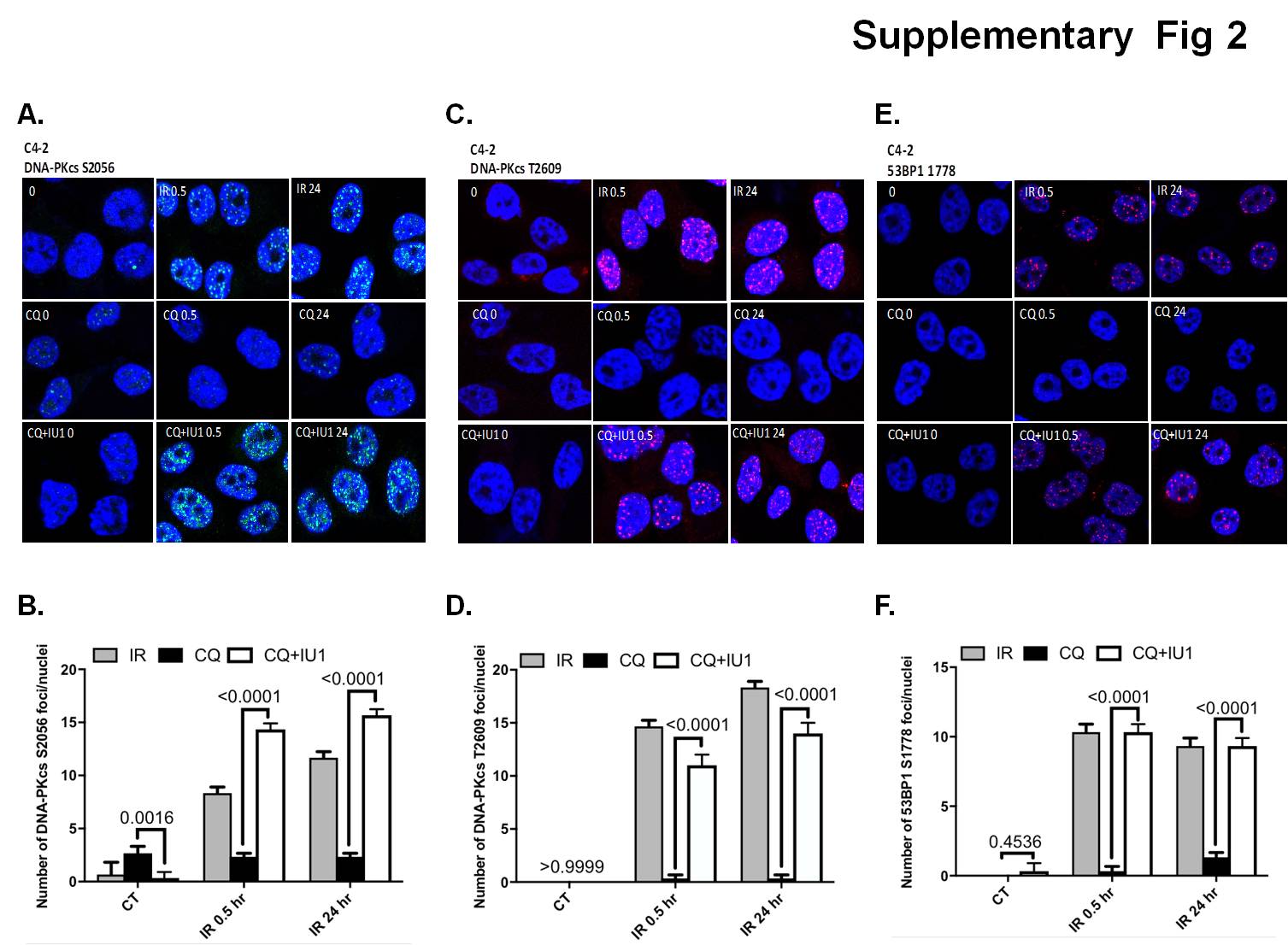

Supplement: gkz1103_Supplemental_Files [file gkz1103_supplemental_files.zip › S2.jpg]

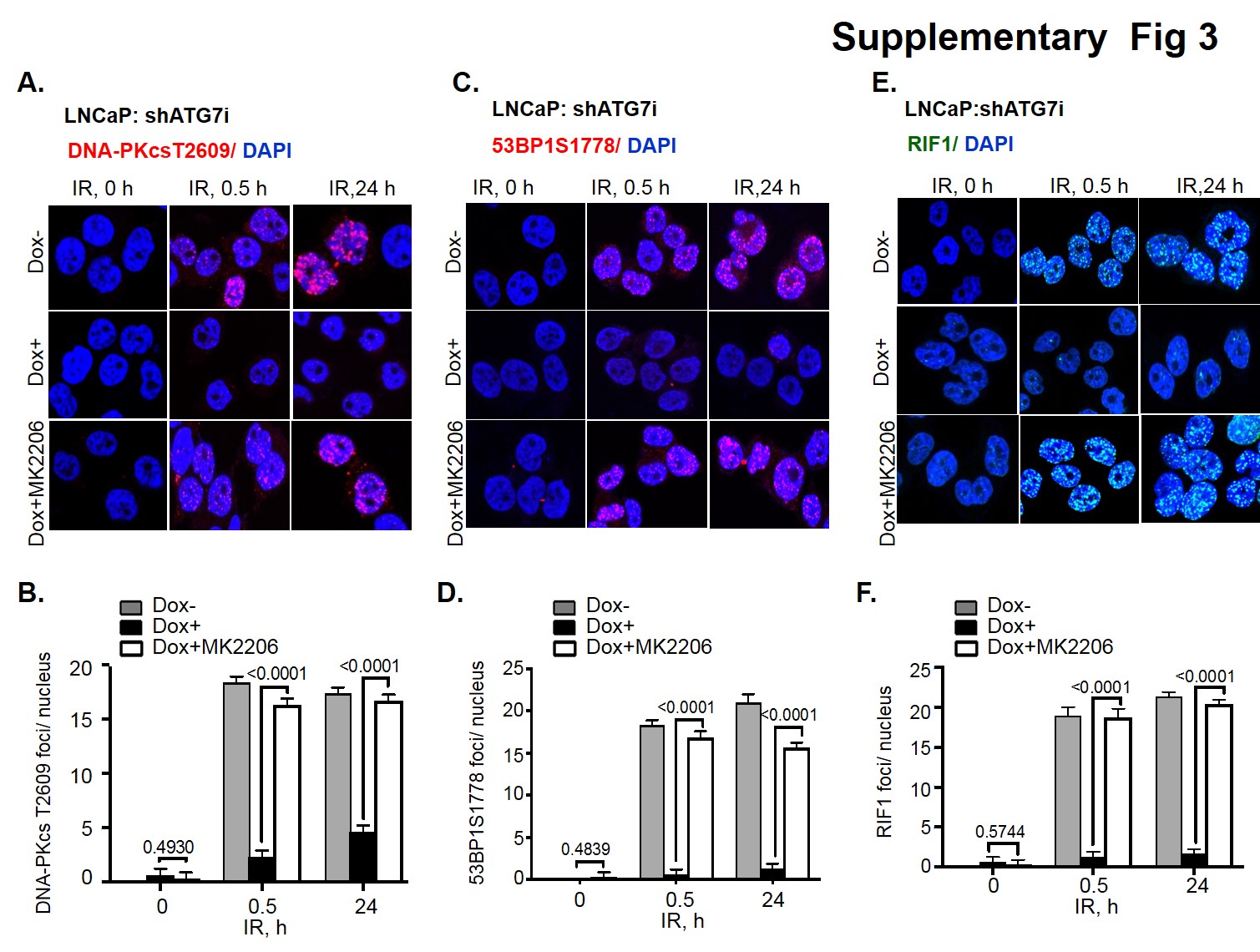

Supplement: gkz1103_Supplemental_Files [file gkz1103_supplemental_files.zip › S3.jpg]

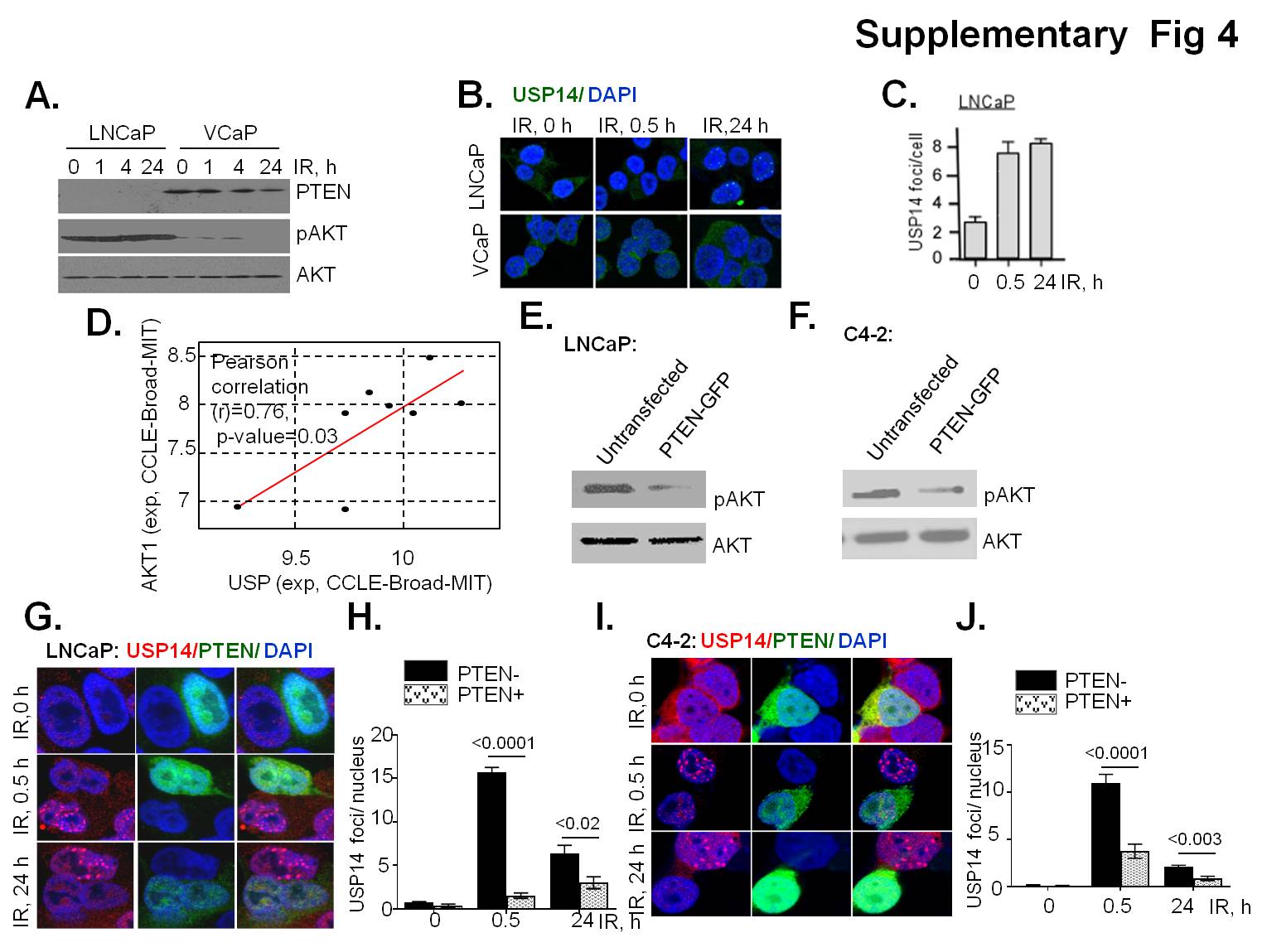

Supplement: gkz1103_Supplemental_Files [file gkz1103_supplemental_files.zip › S4.jpg]
